# 4,6-Dichloro-5-methoxy­pyrimidine

**DOI:** 10.1107/S1600536810001637

**Published:** 2010-01-20

**Authors:** Hoong-Kun Fun, Chin Sing Yeap, C. S. Chidan Kumar, H. S. Yathirajan, M. S. Siddegowda

**Affiliations:** a X-ray Crystallography Unit, School of Physics, Universiti Sains Malaysia, 11800 USM, Penang, Malaysia; b Department of Studies in Chemistry, University of Mysore, Manasagangotri, Mysore 570 006, India

## Abstract

The mol­ecule of the title compound, C_5_H_4_Cl_2_N_2_O, is close to being planar (r.m.s. deviation = 0.013 Å), apart from the C atom of the meth­oxy group, which deviates by 1.082 (2) Å from the mean plane of the other atoms. In the crystal, short Cl⋯N contacts [3.0940 (15) and 3.1006 (17) Å] generate a three-dimensional framework.

## Related literature

For background to the importance of pyrimidines and analogous compounds in pharmaceutical and biological fields, see: Townsend & Drach (2002*a* ▶,*b* ▶). For related structures, see: Bukhari *et al.* (2008 ▶, 2009 ▶); Fun *et al.* (2006 ▶, 2008 ▶)); Yathirajan *et al.* (2007 ▶); Zhao *et al.* (2009 ▶). For the stability of the temperature controller used for the data collection, see: Cosier & Glazer (1986 ▶).
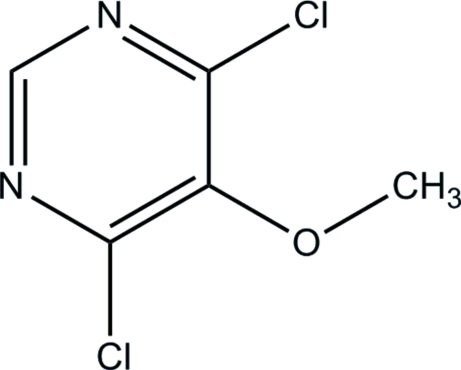


## Experimental

### 

#### Crystal data

C_5_H_4_Cl_2_N_2_O
*M* *_r_* = 179.00
Orthorhombic, 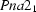

*a* = 13.6545 (19) Å
*b* = 3.9290 (6) Å
*c* = 13.0275 (18) Å
*V* = 698.91 (17) Å^3^
*Z* = 4
Mo *K*α radiation
μ = 0.85 mm^−1^
*T* = 100 K
0.29 × 0.20 × 0.09 mm

#### Data collection

Bruker APEX Duo CCD diffractometer
Absorption correction: multi-scan (*SADABS*; Bruker, 2009 ▶) *T*_min_ = 0.787, *T*_max_ = 0.926
4505 measured reflections
1520 independent reflections
1415 reflections with *I* > 2σ(*I*)
*R*_int_ = 0.024

#### Refinement

*R*[*F* ^2^ > 2σ(*F* ^2^)] = 0.024
*wR*(*F* ^2^) = 0.054
*S* = 1.08
1520 reflections
92 parameters
1 restraint
H-atom parameters constrained
Δρ_max_ = 0.27 e Å^−3^
Δρ_min_ = −0.19 e Å^−3^
Absolute structure: Flack (1983 ▶), 459 Friedel pairs
Flack parameter: −0.02 (6)

### 

Data collection: *APEX2* (Bruker, 2009 ▶); cell refinement: *SAINT* (Bruker, 2009 ▶); data reduction: *SAINT*; program(s) used to solve structure: *SHELXTL* (Sheldrick, 2008 ▶); program(s) used to refine structure: *SHELXTL*; molecular graphics: *SHELXTL*; software used to prepare material for publication: *SHELXTL* and *PLATON* (Spek, 2009 ▶).

## Supplementary Material

Crystal structure: contains datablocks global, I. DOI: 10.1107/S1600536810001637/hb5305sup1.cif

Structure factors: contains datablocks I. DOI: 10.1107/S1600536810001637/hb5305Isup2.hkl

Additional supplementary materials:  crystallographic information; 3D view; checkCIF report

## Figures and Tables

**Fig. 1.**
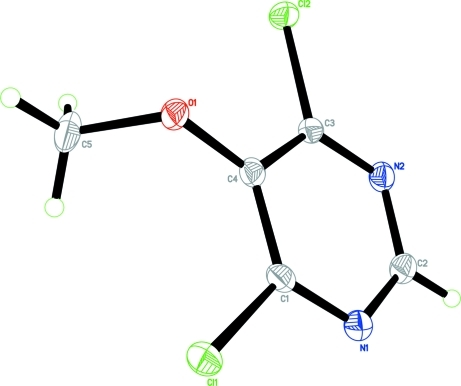
The molecular structure of (I) with 50% probability ellipsoids for non-H atoms.

**Fig. 2.**
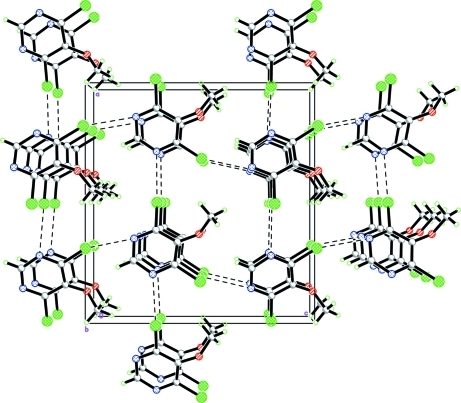
The crystal packing of (I), viewed down the b axis, showing the short contacts (dashed lines) linking the molecules into a three-dimensional framework.

## supplementary crystallographic information

### Comment

The importance of pyrimidines and analogous compounds in pharmaceutical and biological fields is well known (Townsend *et al.*, 2002*a*,*b*)). The crystal structures of 4-(4-bromophenyl)-6-(4-chlorophenyl)pyrimidin-2-ylamine (Bukhari *et al.*, 2009), 4-(4-fluorophenyl)-6-(2-furyl)pyrimidin-2-amine (Bukhari *et al.*, 2008), 2-amino-4,6-dichloropyrimidine (Fun *et al.*, 2008), 4,6-diphenylpyrimidin-2-ylamine (Fun *et al.*, 2006), 5-bromopyrimidin-2(1*H*)-one (Yathirajan *et al.*, 2007) and 4-(4-chlorophenyl)-6-(methylsulfanyl)pyrimidin-2-amine (Zhao *et al.*, 2009) have been reported.  We now report the structure of the title compound, (I).

The geometrical parameters of the title compound (Fig. 1) are comparable to those related structures. In the crystal structure (Fig. 2), molecules are linked into chains by short Cl1···N2 interaction of 3.0940 (15) Å, symmetry code: -1/2 + *x*, 1/2 - *y*, *z*, along the *a* axis. The short Cl2···N1 interaction of 3.1006 (17) Å, symmetry code: 3/2 - *x*, 1/2 + *y*, -1/2 + *z* linked these chains into a three-dimensional framework.

### Experimental

The title compound was obtained as a gift sample from *R. L*. Fine Chem., Bangalore, India. The compound was used without further purification. Colourless blocks of (I) were obtained from the slow evaporation of an acetonitrile solution (m.p.: 313–315 K).

### Refinement

All hydrogen atoms were positioned geometrically with a riding model with C–H = 0.93–0.96 Å and *U*_iso_(H) = 1.2 and 1.5 *U*_eq_(C). A rotating-group model was applied for the methyl groups.

### Crystal data

**Table:**

C_5_H_4_Cl_2_N_2_O	*F*(000) = 360
*M**_r_* = 179.00	*D*_x_ = 1.701 Mg m^−^^3^
Orthorhombic, *P**n**a*2_1_	Mo *K*α radiation, λ = 0.71073 Å
Hall symbol: P 2c -2n	Cell parameters from 1997 reflections
*a* = 13.6545 (19) Å	θ = 3.0–32.2°
*b* = 3.9290 (6) Å	µ = 0.85 mm^−^^1^
*c* = 13.0275 (18) Å	*T* = 100 K
*V* = 698.91 (17) Å^3^	Block, colourless
*Z* = 4	0.29 × 0.20 × 0.09 mm

### Data collection

**Table:**

Bruker APEX Duo CCD diffractometer	1520 independent reflections
Radiation source: fine-focus sealed tube	1415 reflections with *I* > 2σ(*I*)
graphite	*R*_int_ = 0.024
φ and ω scans	θ_max_ = 30.0°, θ_min_ = 3.0°
Absorption correction: multi-scan (*SADABS*; Bruker, 2009)	*h* = −19→17
*T*_min_ = 0.787, *T*_max_ = 0.926	*k* = −5→4
4505 measured reflections	*l* = −18→13

### Refinement

**Table:**

Refinement on *F*^2^	Secondary atom site location: difference Fourier map
Least-squares matrix: full	Hydrogen site location: inferred from neighbouring sites
*R*[*F*^2^ > 2σ(*F*^2^)] = 0.024	H-atom parameters constrained
*wR*(*F*^2^) = 0.054	*w* = 1/[σ^2^(*F*_o_^2^) + (0.0274*P*)^2^ + 0.0046*P*] where *P* = (*F*_o_^2^ + 2*F*_c_^2^)/3
*S* = 1.08	(Δ/σ)_max_ = 0.001
1520 reflections	Δρ_max_ = 0.27 e Å^−^^3^
92 parameters	Δρ_min_ = −0.19 e Å^−^^3^
1 restraint	Absolute structure: Flack (1983), 459 Friedel pairs
Primary atom site location: structure-invariant direct methods	Flack parameter: −0.02 (6)

### Special details

**Table:**

Experimental. The crystal was placed in the cold stream of an Oxford Cyrosystems Cobra open-flow nitrogen cryostat (Cosier & Glazer, 1986) operating at 100.0 (1) K.
Geometry. All e.s.d.'s (except the e.s.d. in the dihedral angle between two l.s. planes) are estimated using the full covariance matrix. The cell e.s.d.'s are taken into account individually in the estimation of e.s.d.'s in distances, angles and torsion angles; correlations between e.s.d.'s in cell parameters are only used when they are defined by crystal symmetry. An approximate (isotropic) treatment of cell e.s.d.'s is used for estimating e.s.d.'s involving l.s. planes.
Refinement. Refinement of *F*^2^ against ALL reflections. The weighted *R*-factor *wR* and goodness of fit *S* are based on *F*^2^, conventional *R*-factors *R* are based on *F*, with *F* set to zero for negative *F*^2^. The threshold expression of *F*^2^ > σ(*F*^2^) is used only for calculating *R*-factors(gt) *etc*. and is not relevant to the choice of reflections for refinement. *R*-factors based on *F*^2^ are statistically about twice as large as those based on *F*, and *R*- factors based on ALL data will be even larger.

### Fractional atomic coordinates and isotropic or equivalent isotropic displacement parameters (Å^2^)

**Table:**

	*x*	*y*	*z*	*U*_iso_*/*U*_eq_	
Cl1	1.00227 (3)	0.39313 (10)	0.31491 (4)	0.01880 (10)	
Cl2	0.68657 (3)	−0.13063 (10)	0.50590 (3)	0.01823 (10)	
O1	0.87265 (8)	0.2620 (3)	0.49779 (10)	0.0158 (2)	
N1	0.85636 (12)	0.0895 (4)	0.22384 (12)	0.0173 (3)	
N2	0.71682 (10)	−0.1454 (3)	0.30807 (13)	0.0164 (3)	
C1	0.89051 (11)	0.1878 (4)	0.31413 (16)	0.0146 (3)	
C2	0.77002 (14)	−0.0714 (5)	0.22517 (14)	0.0176 (4)	
H2A	0.7446	−0.1382	0.1621	0.021*	
C3	0.75310 (13)	−0.0416 (4)	0.39696 (13)	0.0134 (3)	
C4	0.84177 (14)	0.1346 (4)	0.40704 (14)	0.0136 (3)	
C5	0.94496 (15)	0.0608 (5)	0.55200 (16)	0.0225 (4)	
H5A	0.9966	−0.0013	0.5057	0.034*	
H5B	0.9147	−0.1413	0.5786	0.034*	
H5C	0.9715	0.1917	0.6077	0.034*	

### Atomic displacement parameters (Å^2^)

**Table:**

	*U*^11^	*U*^22^	*U*^33^	*U*^12^	*U*^13^	*U*^23^
Cl1	0.01301 (17)	0.02330 (19)	0.02010 (18)	−0.00290 (14)	0.00081 (16)	0.0021 (2)
Cl2	0.01565 (17)	0.02443 (19)	0.01463 (17)	−0.00177 (15)	0.00211 (17)	0.00248 (19)
O1	0.0159 (5)	0.0185 (5)	0.0130 (5)	0.0029 (4)	−0.0029 (6)	−0.0044 (6)
N1	0.0148 (7)	0.0222 (8)	0.0150 (7)	0.0019 (6)	−0.0012 (6)	−0.0009 (6)
N2	0.0152 (6)	0.0182 (7)	0.0159 (7)	0.0014 (5)	−0.0030 (7)	−0.0010 (6)
C1	0.0114 (6)	0.0150 (7)	0.0174 (7)	0.0020 (5)	0.0012 (8)	0.0010 (7)
C2	0.0172 (9)	0.0213 (10)	0.0145 (8)	0.0015 (7)	−0.0028 (7)	−0.0019 (7)
C3	0.0127 (8)	0.0144 (8)	0.0130 (7)	0.0017 (6)	0.0005 (7)	0.0016 (6)
C4	0.0133 (8)	0.0133 (7)	0.0142 (8)	0.0030 (5)	−0.0015 (6)	−0.0003 (6)
C5	0.0261 (10)	0.0243 (9)	0.0170 (8)	0.0052 (7)	−0.0097 (8)	−0.0013 (8)

### Geometric parameters (Å, °)

**Table:**

Cl1—C1	1.7262 (16)	N2—C2	1.334 (2)
Cl2—C3	1.7210 (19)	C1—C4	1.397 (3)
O1—C4	1.351 (2)	C2—H2A	0.9300
O1—C5	1.449 (2)	C3—C4	1.401 (3)
N1—C1	1.323 (2)	C5—H5A	0.9600
N1—C2	1.338 (2)	C5—H5B	0.9600
N2—C3	1.324 (2)	C5—H5C	0.9600
			
C4—O1—C5	115.92 (13)	C4—C3—Cl2	118.65 (14)
C1—N1—C2	115.91 (16)	O1—C4—C1	123.64 (16)
C3—N2—C2	115.93 (14)	O1—C4—C3	122.32 (16)
N1—C1—C4	123.97 (15)	C1—C4—C3	113.86 (16)
N1—C1—Cl1	116.95 (14)	O1—C5—H5A	109.5
C4—C1—Cl1	119.08 (14)	O1—C5—H5B	109.5
N2—C2—N1	126.41 (17)	H5A—C5—H5B	109.5
N2—C2—H2A	116.8	O1—C5—H5C	109.5
N1—C2—H2A	116.8	H5A—C5—H5C	109.5
N2—C3—C4	123.89 (16)	H5B—C5—H5C	109.5
N2—C3—Cl2	117.46 (14)		
			
C2—N1—C1—C4	−0.4 (2)	N1—C1—C4—O1	−173.85 (16)
C2—N1—C1—Cl1	179.51 (12)	Cl1—C1—C4—O1	6.2 (2)
C3—N2—C2—N1	1.5 (2)	N1—C1—C4—C3	1.4 (2)
C1—N1—C2—N2	−1.1 (3)	Cl1—C1—C4—C3	−178.53 (12)
C2—N2—C3—C4	−0.3 (2)	N2—C3—C4—O1	174.29 (15)
C2—N2—C3—Cl2	179.80 (13)	Cl2—C3—C4—O1	−5.8 (2)
C5—O1—C4—C1	−85.2 (2)	N2—C3—C4—C1	−1.0 (2)
C5—O1—C4—C3	99.96 (19)	Cl2—C3—C4—C1	178.89 (12)
